# Diabetes and nephrotic syndrome: Answers

**DOI:** 10.1007/s00467-016-3560-9

**Published:** 2016-12-23

**Authors:** Rodney D Gilbert, Edward Hind, Bhumita Vadgama

**Affiliations:** 1 Southampton Children’s Hospital and University of Southampton School of Medicine, Tremona Road, Southampton, SO16 6YD UK; 2 Hampshire Hospitals NHS Foundation Trust, Basingstoke, Hampshire, UK; 3 Department of Cellular Pathology, University Hospital Southampton, Southampton, UK

**Keywords:** Diabetes, Nephrotic syndrome, Nephrotic syndrome genetic causes, MIDD

## Answers to questions

### Question 1: How are the diabetes and renal disease connected to each other, if at all?

In principle, when a patient has multiple signs, symptoms or laboratory abnormalities they can be explained in one of four ways:

1. All the problems may be features of a single disease.
2. Some of the problems may be complications or side effects of treatment given for one or more problems or diseases.
3. One or more problems may be a complication of another condition.
4. The patient may have multiple, unrelated diseases.

The first choice in formulating a differential diagnosis is usually to find a single condition to explain everything. In a patient with diabetes and proteinuria, the initial thought is often diabetic nephropathy. However, diabetic patients are more likely to have conditions other than diabetic nephropathy [1]. This is particularly true in patients with short duration of diabetes and good metabolic control. Our patient has diabetes which is somewhat atypical for type 1 diabetes with negative auto-antibodies and an unusually long “honeymoon period” with very low glycated haemoglobin (HbA_1_C) levels [2]. The renal biopsy showed focal segmental glomerulosclerosis (FSGS), and she has other clinical features, including short stature, poor effort tolerance and abdominal pain. One needs to consider genetic causes of diabetes that may account for some or all of these features. The patient had received large doses of analgesics, but the renal disease is unusual for analgesic nephropathy which typically presents with papillary necrosis and reduced glomerular filtration rate (GFR). The association with drugs other than phenacetin remains speculative [3]. Finally, the patient could have two or more unrelated conditions.

### Question 2: What further investigations should be undertaken?

It would be appropriate to undertake genetic testing for diabetes and possibly for genetic causes of nephrotic syndrome.

### Question 3: What is the diagnosis?

Genetic testing revealed a mutation in mitochondrial DNA, m.3243A>G, which is the most common cause of maternally inherited diabetes and deafness (MIDD), a mitochondrial disorder characterized by maternally transmitted diabetes and sensorineural deafness. She was also found to be heterozygous for a missence variant of *NPHS1*, c.2746G>T; p.(Ala916Ser). The patient therefore has MIDD, and the renal disease may be aggravated by the *NPHS1* variant.

## Discussion

Maternally inherited diabetes and deafness was first described in 1992 [4]. It is caused in the great majority of cases by an adenine to guanine substitution at position 3243 of the mitochondrial DNA (m.3243A>G) encoding the gene for leucine transfer RNA. MIDD is frequently misdiagnosed as type 1 or type 2 diabetes, depending on the age of the patient and mode of presentation. About 1% of cases of diabetes are thought to be caused by mutations in mitochondrial DNA, and the m.3243A>G mutation accounts for about 85% of these [5].

The key clinical features are diabetes and deafness and their presence in maternal relatives. As the case of our patient demonstrates, these are not always present. The diabetes usually presents insidiously, but in 20% of cases it presents acutely, with ketoacidosis present in 8% of the latter cases [6]. About 75% of diabetic patients with the m.3243A>G mutation have deafness, which is sensorineural and due to cochlear disease [7].

Renal disease is common in patients with MIDD, especially females. In one series of 74 patients with MIDD, proteinuria was greater in these patients than in control diabetic patients, and chronic kidney disease (CKD) stage 3 or greater was four-to sixfold higher despite lower HbA_1_C, lower blood pressure and a 3.7-fold lower incidence of diabetic retinopathy than controls [8]. The commonest histological pattern in patients undergoing renal biopsy is FSGS, but tubulointerstitial disease and renal cysts have also been described. The combination of nephropathy and deafness may lead to a mistaken diagnosis of Alport syndrome [9].

Several other features present in this patient are also explained by the m.3243A>G mutation, including short stature, which is common and caused by a deficiency of growth hormone–releasing hormone [10, 11]. Gastro-intestinal complaints are also common, especially constipation and pseudo-obstruction [5]. The inability to play competitive football is likely to reflect impaired energy utilization and skeletal myopathy. Cardiomyopathy, retinal macular dystrophy and stroke, features not present in our patient, are also described.

Our patient had early onset of several features. This is likely to reflect high levels of heteroplasmy for the mutant mitochondrial DNA. The severity and early onset of renal disease may also be due to the presence of a heterozygous mutation in *NPHS1*. This gene codes for nephrin, an important component of the glomerular slit diaphragm, and homozygous mutations cause Finnish type congenital nephrotic syndrome.

A diagnosis of MIDD has several important implications for treatment. Metformin should not be used for treating the diabetes because of the increased risk of lactic acidosis. Antibiotics such as tetracyclines and chloramphenicol which interfere with mitochondrial function should also be avoided. Coenzyme Q10 has been shown to be beneficial in preventing hearing loss and delaying progression of diabetes [12]. In patients who progress to end stage renal failure, great caution should be exercised in using maternal relatives as kidney donors.

## Learning points

- Islet cell and glutamic acid decarboxylase (GAD) antibodies should be tested in diabetic patients with atypical disease behaviour or features. Genetic testing should be considered in patients who test negative for islet cell and GAD antibodies. A probability calculator for monogenic diabetes is available at http://www.diabetesgenes.org/content/mody-probability-calculator .
- Young diabetic patients with nephropathy and good diabetic control are more likely to have disease other than diabetic nephropathy, especially if there is no diabetic retinopathy.
- MIDD may cause renal failure and deafness which may be misdiagnosed as Alport syndrome.

## References

[1] Sharma SG, Bomback AS, Radhakrshnan J, Herlitz LC, Stokes MB, Markowitz GS, D’Agati VD (2013). The modern spectrum of renal biopsy findings in patients with diabetes. Clin J Am Soc Nephrol.

[2] Lombardo F, Valenzise M, Wasniewska M, Messina MF, Ruggeri C, Arrigo T, De Luca F (2002). Two-year prospective evaluation of the factors affecting honeymoonfrequency and duration in children with insulin dependent diabetes mellitus: the key role of age at diagnosis. Diabetes Nutr Metab.

[3] Yaxley J, Litfin T (2016). Non-steroidal anti-inflammatories and the development of analgesic nephropathy: a systematic review. Renal Fail.

[4] van den Ouweland JM, Lemkes HH, Ruitenbeek W, Sandkujl LA, de Vijlder MF, Struyvenberg PA, van de Kamp JJ, Maassen JA (1992). Mutation in mitochondrial tRNA Leu(UUR) gene in a large pedigree with maternally transmitted Type 2 diabetes and deafness. Nat Genet.

[5] Murphy R, Turnbull TM, Walker M, Hattersley AT (2008). Clinical features, diagnosis and management of maternally inherited diabetes and deafness (MIDD) associated with the 3243A>G mitochondrial point mutation. Diabetic Med.

[6] Guillausseau PJ, Dubois-Laforge D, Massin P, Laloi-Michelin M, Bellanne-Chantelot C, Gin H, Bellanné-Chantelot C, Gin H, Bertin E, Blickle JF, Bauduceau B, Bouhanick B, Cahen-Varsaux J, Casanova S, Charpentier G, Chedin P, Derrien C, Grimaldi A, Guerci B, Kaloustian E, Lorenzini F, Murat A, Olivier F, Paques M, Paquis-Flucklinger V, Tielmans A, Vincenot M, Vialettes B, Timsit J, GEDIAM, Mitochondrial Diabetes French Study Group (2004). Heterogeneity of diabetes phenotype in patients with 3243-bp mutation of mitochondrial DNA (maternally inherited diabetes and deafness or MIDD). Diabetes Metab.

[7] Yamasoba T, Oka T, Tsukuda K, Nakamura M, Kaga K (1996). Auditory findings in patients with maternally inherited diabetes and deafness harboring a point mutation in the mitochondrial transfer RNA Leu(UUR) gene. Laryngoscope.

[8] Massin P, Dubois-Laforgue D, Meas T, Laloi-Michelin M, Gin H, Bauduceau C, Bellanné-Chantelot C, Bertin E, Blickle JF, Bouhanick B, Cahen-Varsaux J, Casanova S, Charpentier G, Chedin P, Dupuy O, Grimaldi A, Guerci B, Kaloustian E, Lecleire-Collet A, Lorenzini F, Murat A, Narbonne H, Olivier F, Paquis-Flucklinger V, Virally M, Vincenot M, Vialettes B, Timsit J, Guillausseau PJ, GEDIAM (Mitochondrial Diabetes French Study Group (2008). Retinal and renal complications in patients with a mutation of mitochondrial DNA at position 3,243 (maternally inherited diabetes and deafness). A case–control study. Diabetologica.

[9] Jansen JJ, Maassen JA, van der Woude FJ, Lemmink HAJ, van den Ouweland JMW, t Hart LM, Smeets HJM, Bruijn JA, Lemkes HPJ (1997). Mutation in mitochondrial tRNA^Leu(UUR)^ gene associated with progressive kidney disease. J Am Soc Nephrol.

[10] Ohkubo K, Yamano A, Nagashima M, Mori Y, Anzai K, Akehi Y, Nomiyama R, Asano T, Urae A, Ono J (2001). Mitochondrial gene mutations in the tRNA Leu(UUR) region and diabetes: prevalence and clinical phenotypes in Japan. Clin Chem.

[11] Koga Y, Akita Y, Takane N, Sato Y, Kato H (2000). Heterogeneous presentation in A3243G mutation in the mitochondrial tRNA^Leu(UUR)^ gene. Arch Dis Child.

[12] Suzuki S, Hinokio Y, Ohtomo M, Hirai M, Hirai A, Chiba M, Kasuga S, Akai H, Toyota T (1998). The effects of coenzyme Q10 treatment on maternally inherited diabetes and deafness, and mitochondrial DNA 3243 (A to G) mutation. Diabetologia.

